# In Vitro Efficacy of PEI-Derived Lipopolymers in Silencing of Toxic Proteins in a Neuronal Model of Huntington’s Disease

**DOI:** 10.3390/pharmaceutics17060726

**Published:** 2025-05-30

**Authors:** Luis C. Morales, Luv Modi, Saba Abbasi Dezfouli, Amarnath Praphakar Rajendran, Remant Kc, Vaibhavi Kadam, Simonetta Sipione, Hasan Uludağ

**Affiliations:** 1 Department of Chemical and Materials Engineering, University of Alberta, Edmonton, AB T6G 2R3, Canada; lmodi@ualberta.ca (L.M.); sabbasid@ualberta.ca (S.A.D.); amarnath@ualberta.ca (A.P.R.); remant@ualberta.ca (R.K.); huludag@ualberta.ca (H.U.); 2 Department of Pharmacology, University of Alberta, Edmonton, AB T6G 2R3, Canada; vaibhavi@ualberta.ca (V.K.); ssipione@ualberta.ca (S.S.); 3 Faculty of Pharmacy & Pharmaceutical Sciences, University of Alberta, Edmonton, AB T6G 2R3, Canada; 4 Neuroscience and Mental Health Institute, University of Alberta, Edmonton, AB T6G 2R3, Canada

**Keywords:** Huntington’s disease, lipopolymers, gene silencing

## Abstract

**Background:** Huntington’s Disease (HD) is a neurodegenerative disorder caused by an abnormal extension of a CAG repeat stretch located in the exon 1 of the *HTT* (IT15) gene, leading to production of a mutated and misfolded Huntingtin protein (muHTT) with an abnormally elongated polyglutamine (polyQ) region. This mutation causes muHTT to oligomerize and aggregate in the brain, particularly in the striatum and cortex, causing alterations in intracellular trafficking, caspase activation, and ganglioside metabolism, ultimately leading to neuronal damage and death and causing signs and symptoms such as chorea and cognitive dysfunction. Currently, there is no available cure for HD patients; hence, there is a strong need to look for effective therapies. **Methods:** This study aims to investigate the efficacy of siRNA-containing nano-engineered lipopolymers in selectively silencing the *HTT* expression in a neuronal model expressing a chimeric protein formed by the human mutated exon 1 of the *HTT* gene, tagged with GFP. Toxicity of lipopolymers was assessed using MTT assay, while efficacy of silencing was monitored using qRT-PCR, as well as Western blotting/flow cytometry. Changes in muHTT-GFP aggregation were observed using fluorescence microscopy and image analyses. **Results:** Here, we show that engineered lipopolymers can be used as delivery vehicles for specific siRNAs, decreasing the transcription of the mutated gene, as well as the muHTT protein production and aggregation, with Leu-Fect C being the most effective candidate amongst the assessed lipopolymers. **Conclusions:** Our findings have profound implications for genetic disorder therapies, highlighting the potential of nano-engineered materials for silencing mutant genes and facilitating molecular transfection across cellular barriers. This successful in vitro study paves the way for future in vivo investigations with preclinical models, offering hope for previously considered incurable diseases such as HD.

## 1. Introduction

Huntington’s Disease (HD) is a fatal genetic neurodegenerative disease clinically characterized by lack of coordination, severe and involuntary jerky movements known as chorea, as well as neurocognitive dysfunction, depression, anxiety, amongst others signs and symptoms [1,2,3,4,5]. HD is caused by a genetic mutation in the exon 1 of the *HTT* (IT15) gene, which results in the production of a mutated and abnormal Huntingtin protein (muHTT) [5,6]. The mutation responsible for HD is an expansion of CAG repeat sequences which leads to an extension of the polyglutamine (Q) tract in the muHTT. It has been shown that muHTT is prone to aggregation in oligomers and inclusion bodies, predominantly in the striatum and cortex, causing devastating cell death in those regions, respectively, leading to the onset and worsening of HD symptoms [3,7,8,9]. Currently, there is no cure nor disease-modifying treatment available for HD patients; hence, the foremost need to look for emerging therapies for such a devastating disease.

The pursuit of effective treatments for HD has led to a diverse array of therapeutic strategies targeting the production of muHTT using a plethora of delivery mechanisms, each at varying stages of clinical development. In the preclinical phase, several approaches are under investigation: Takeda and Sangamo Therapeutics are developing TAK-686, an adeno-associated virus (AAV9)-delivered zinc finger protein aimed at allele-selective suppression of *HTT*; Neubase Therapeutics is developing NT100, an antisense oligonucleotide (ASO) aimed at reducing HTT levels; Spark Therapeutics is investigating RNAi approaches targeting HTT with their investigational drug SPK-10001; and Atalanta Therapeutics, in partnership with Biogen, is working on siRNA-based therapies for HTT reduction [10]. Several studies have investigated the therapeutic potential of AAV5-miHTT, an adeno-associated viral vector delivering microRNAs that target HTT mRNA in HD mouse models. Thomson et al. (2023), Caron et al. (2020), and Sogorb-Gonzalez et al. (2024) demonstrated that intrastriatal administration of AAV5-miHTT resulted in dose-dependent reductions in mutant HTT in both the striatum and cortex [11,12,13]. High-dose AAV5-miHTT treatment in Q175FDN mice alleviated hippocampal atrophy and partially normalized neurochemical alterations. In contrast, Caron et al. observed that high-dose treatment in humanized Hu128/21 mice led to astrogliosis and motor coordination impairments, emphasizing the importance of precise dosing to balance therapeutic benefits and adverse effects. Sogorb-Gonzalez et al. targeted exon 1 of the HTT gene, achieving effective reduction in both full-length mutant HTT and pathogenic HTT exon 1 proteins [13], suggesting that simultaneous lowering of both could offer enhanced therapeutic efficacy. Zhang et al. (2021) developed a synthetic biology-based approach for delivering siRNAs to the central nervous system in HD therapy. Their strategy involved engineering a genetic circuit that reprogrammed hepatocytes to produce exosomes loaded with siRNA targeting mutant HTT. These exosomes, modified with a rabies virus glycoprotein tag, facilitated crossing the blood–brain barrier (BBB) and targeted neurons in the cortex and striatum. In mouse models of HD, this approach led to significant reductions in mHTT protein levels and aggregates, resulting in improved motor functions and alleviated neuropathology [14].

Regarding potential siRNA/miRNA-based potential therapeutic approaches, several promising candidates are progressing into clinical trials. UniQure is conducting Phase 1/2 trials for AMT-130, an AAV5-delivered microRNA therapy targeting *HTT*, while PTC Therapeutics is advancing PTC518, an oral mRNA splicing modulator, in Phase 2 trials (PIVOT-HD). Both treatments have received U.S. FDA Fast Track designation [15]. Alnylam Pharmaceuticals, in collaboration with Regeneron Pharmaceuticals, is advancing ALN-HTT, a siRNA-based therapy targeting HTT expression, and VICO Therapeutics is advancing VO659, an ASO with potential allele selectivity for HTT suppression. Additionally, Wave Life Sciences initiated Phase 1b/2a trials for WVE-120101 and WVE-120102, which target specific HTT mutations but were discontinued due to suboptimal results [15]. In 2021, a new version of ASO with improved backbone chemistry, WVE-003, was introduced for Phase 1b/2a clinical trials [10]. In Phase 3, Roche, in collaboration with Ionis Pharmaceuticals, explored Tominersen (RG6042), an ASO designed to reduce HTT production, although significant concerns over an unfavorable benefit/risk profile were noted [15]. These diverse therapeutic strategies reflect the ongoing commitment to developing effective treatments for HD, each employing unique mechanisms and delivery methods at different stages of clinical progression [10].

Therapies relying on siRNA have emerged as a promising approach for targeting mutated HD cells with high specificity and effectiveness by selectively silencing the disease-causing gene. The siRNAs utilize the endogenous RNA Interference (RNAi) pathway to disrupt the mRNA coded proteins that lead to undesirable actions [4,5]. As long as the target mRNA sequence is known, siRNAs could be designed at will to interrupt the mRNA-coded proteins. Nonetheless, siRNA transport to target cells remains a major difficulty due to the large size of the siRNAs, their hydrophilicity, negative charge (which antagonizes with lipophilic cell membranes), and sensitivity to breakdown by host enzymes such as the systemic nucleases. To counter this effect, the anionic siRNAs need to be delivered using an effective carrier. Both viral and non-viral vectors have been successfully employed for siRNA delivery. Viral vectors, such as adenoviruses and lentiviruses, offer high transduction efficiency but raise concerns over insertional mutagenesis and immunogenicity. Non-viral vectors, in contrast, offer advantages in engineering flexibility and lower production costs, though they face challenges related to lower transfection efficiency and cytotoxicity [16]. It is essential to consider that nanoparticle-based siRNA delivery systems can affect pharmacological activity beyond their intended targets. At the nanoscale, materials previously considered inert may exhibit increased reactivity due to a larger surface area, potentially leading to “nanotoxicity”. While these systems can improve RNAi, they must be designed to avoid interfering with normal cellular functions and minimize toxicity [17].

Cationic polymers, particularly polyethyleneimine (PEI), have been widely used for their ability to electrostatically bind nucleic acids, facilitating cellular uptake and protecting siRNA from degradation. Their high transfection efficiency is largely attributed to the “proton sponge” effect, wherein the polymer’s amine groups buffer the endosomal environment, leading to osmotic swelling and subsequent release of the siRNA cargo into the cytoplasm [18]. However, PEI’s high molecular weight can cause cytotoxicity, necessitating the use of low molecular weight PEI for safer and more effective formulations. Modifications to the PEI backbone, such as lipid substitutions, have been shown to enhance the uptake of siRNA/PEI complexes in various models, likely due to improved compatibility with cellular membranes. These lipid-modified PEI derivatives not only improve transfection efficiency but also maintain lower cytotoxic profiles, offering a promising avenue for therapeutic siRNA delivery [19,20,21]. The lipid-modified PEI lipopolymers largely remain to be explored in neurodegenerative disease models.

In this study, we show that a series of proprietary lipid-modified PEI lipopolymers effectively delivered HTT-specific siRNAs, leading to a reduction in mutant transgene transcription, as well as decreased muHTT production and aggregation in a cellular model where the exon 1 human muHTT is constitutively overexpressed. These results underscore the potential of nano-engineered materials in genetic disorder therapies by enabling targeted gene silencing and enhancing transfection in in vitro models or HD. They serve as the foundation of future in vivo studies using preclinical models of HD, with the hope for developing promising therapeutic strategies against neurodegenerative diseases such as HD.

## 2. Materials and Methods

### 2.1. Lipid-Substituted PEI-Derived Lipopolimers and Trans-Booster Additive

The lipopolimers of the Leu-Fect series including Leu-Fect A, B, and C are chemically synthesized by RJH Biosciencies Inc (Edmonton, AB, Canada) by substituting the amine groups of low molecular weight, branched PEI (2100 Da) with highly biocompatible lipids. The exact nature of the lipids as well as the substitution ratios are brand-protected and their intellectual property solely belongs to RJH Biosciences. Trans-Booster additive is a water-soluble high-molecular-weight anionic polymer, and the exact nature of it is also brand-protected.

### 2.2. Materials

The 3-[4,5-Dimethylthiazol-2-yl]-2,5-diphenyltetrazolium bromide (MTT), Dulbecco’s Phosphate-Buffered Saline (PBS), cell-culture grade Penicillin/Streptomycin solution (100X), Dulbecco’s Modified Eagle Medium F12 (DMEM/F12), branched 25 kDa polyethyleneimine (bPEI), 2.5% Bovine Serum Albumin (BSA) solution, and dimethyl sulfoxide (DMSO) were obtained from MiliporeSigma (Saint Louis, MO, USA). GFP-siRNA, negative control scrambled siRNA, 6-′(FAM)-labeled scrambled siRNA, and muHTT siRNA were obtained from Integrated DNA Technologies Inc. (Coralville, IA, USA). Fetal Bovine Serum (FBS) was obtained from VWR (Radnor, PA, USA). Complete protease inhibitors and PhosStop phosphatase inhibitors were from Roche (Basel, Switzerland). Trypsin-EDTA and TRIZOL reagents were purchased from ThermoFisher Scientific Inc. (Waltham, MA, USA). To create 3.7% formaldehyde for cell fixing, Hank’s Balanced Salt Solution (HBSS) was used to dilute a 37% stock solution, which was purchased from Sigma-Aldrich (St. Louis, MO, USA). Lipofectamine^TM^ RNAiMax was obtained from Thermo-Fisher and used as per manufacturer’s instructions. The antibody for HTT detection (N17) was produced and kindly donated by Dr. Ray Truant (University of British Columbia, Kelowna, BC, Canada).

### 2.3. Cell Culture

Attachment-dependent N2a-97Q cells were generated by stably expressing a construct containing the exon 1 of the human HTT gene with 97 CAG repeats, C-terminally tagged with eGFP into naive mouse N2a cells, followed by selection with 400 µg/mL of neomycin [22]. Cells were maintained in the DMEM containing 10% FBS, 100 U/mL of penicillin, and 100 μg/mL of streptomycin in a humidified atmosphere of 37 °C and 5% CO_2_.

### 2.4. Transfection of siRNAs Using Leu-Fect Lipopolymers

To assess the potency of the lipopolymers in conjunction with siRNAs specifically targeting the human *HTT* gene within the N2a-97Q cell model, a comprehensive set of four functional siRNAs was deployed alongside a reference control (scrambled) siRNA. Three siRNAs were designed to target exon 1 of *HTT*, including the muHTT transgene expressed in N2a-97Q cells, based on existing literature or using bioinformatic tools (namely HTT1 [23], HTT2 [24], HTT3 using GeneScript siRNA design tool). GFP siRNA has been described elsewhere [25]. The siRNA sense and antisense sequences are provided in Table 1.

For the preparation of complexes, the siRNAs were diluted in DMEM without FBS at desired amounts. If Trans-Booster is used in complexes, it is added at a ratio of 1:1 (*w*/*w*) to siRNA amount. Then, the transfection reagents, Leu-Fects, were added to this solution at ratios (*w*/*w*) ranging from 2.5:1 to 15:1, as stated in each experiment. Complex formation was allowed to occur for approximately 30 min at room temperature before the complexes were added directly to the culture medium to a final concentration of 40–60 nM of siRNA.

### 2.5. MTT Assay for Cytotoxicity

The cytotoxicity of complexes was measured by the MTT assay. Briefly, the N2a-97Q cells were seeded on 96-well plates, and after 24 h, cells were treated with the complexes prepared with the Leu-Fect A, Leu-Fect B, Leu-Fect C, branched PEI, as well as Lipofectamine^TM^ RNAiMAX. Forty-eight hours after the treatment, 100 μL of MTT solution (5 mg/mL) was added onto the cells and incubated for 1.5 h. The medium was then removed after formation of formazan crystals that were solubilized in pure DMSO. Absorbance was measured with a microplate reader at a wavelength of 570 nm. The proportion of cell survival was calculated based on the absorbance of untreated cells.

### 2.6. Flow Cytometry Analysis

The N2a-97Q cells were seeded in 48-well plates (Corning, Corning, NY, USA) and treated with complexes prepared with Leu-Fect A, Leu-Fect B, and Leu-Fect C using a carrier/siRNA ratio of 5:1 (*w*/*w*), and with Lipofectamine^TM^ RNAiMAX complexes they were prepared at a ratio of 1:1 and 2:1. Silencing of the muHTT after these treatments was monitored 48 h after transfection. To determine the extent of muHTT silencing, cells were washed once with PBS and then detached using 100 µL of trypsin-EDTA 0.25% solution. The suspension was mixed with 100 µL of paraformaldehyde and analyzed either in an Attune NxT Flow Cytometer (Thermo-Fisher, Waltham, MA, USA) or a BD LSRFortessa^TM^ Cell Analyzer (Beckton-Dickinson, Franklin Lakes, NJ, USA). The percentage of GFP-positive cells and mean intensity fluorescence were plotted for each of the treatments.

### 2.7. Image Analysis of muHTT Aggregates

A subpopulation of N2a-97Q cells displays aggregation of muHTT-GFP protein, characterized by high fluorescence intracellular/perinuclear punctae. To quantify the extent of muHTT-GFP aggregation, N2a-97Q cells were plated in 48-well plates at 21,000 cells/well and then transfected with the siRNA complexes. Forty-eight hours after transfection, cells were washed with PBS and then fixed with 4% *w*/*v* paraformaldehyde. Nuclei were stained using 1 μg/mL DAPI solution. A total of 270 pictures per treatment was taken using an Olympus FX100 inverted fluorescence microscope. Image analysis was performed using NIH ImageJ software (Version 1.54m). Briefly, GFP images were binarized using a threshold containing pixels whose intensity was equal or greater than the 95–99 percentile. Once binarized, the total GFP-positive area and total number of GFP-positive particles was quantified. To account for potential bias due to the number of cells per field, data were normalized over the DAPI-positive area in each microphotograph.

### 2.8. RT-qPCR

The N2a-97Q cells were seeded in a 12-well plate at 65,000 cells/well and allowed to attach for 24 h. Then, cells were transfected with the complexes of Leu-Fect C and Lipofectamine^TM^ RNAiMAX along with 5 corresponding siRNAs (Ctrl, HTT1, HTT2, HTT3, and GFP siRNAs). The Leu-Fect C/siRNA was used at a 5:1 carrier:siRNA ratio. The Lipofectamine^TM^ RNAiMAX/siRNA complexes were used at a ratio of 2:1. After 24 or 48 h, the total RNA was isolated using Trizol reagent and quantified using the Nanodrop Spectrophotometer (Thermo-Fisher, Waltham, MA, USA). cDNA synthesis was carried out using the cDNA synthesis kit (Thermo-Fisher, Waltham, MA, USA) followed by qPCR using the SensiFAST SYBR Hi-Rox Kit (Meridian Bioscience, Cincinnati, OH, USA) on a Step-One-Plus thermocycler (Thermo-Fisher, Waltham, MA, USA). Primers were designed to bind the N17 region of the exon 1 of the HTT gene (FWD: 5′-GACCCTGGAAAAGCTGATGA-3′; REV: 5′-TTGGAAGCTTTTGAGGGACT-3′. Quantitative analyses were conducted using the 2–∆∆Ct method and the mHTT mRNA transcript levels were presented relative to the untreated control. Beta-actin (ACTB) gene was used as the housekeeping gene.

### 2.9. Western Blotting

The N2a-97Q cells were cultured in 6-well plates and treated with different complexes. Forty eight hours after transfection, cells were lysed in RIPA buffer containing complete protease inhibitors and PhosStop phosphatase inhibitors. Total protein concentration cell lysates were measured by the BCA assay (Pierce, Appleton, WI, USA). A total of 25 µg of protein was loaded into 4–20% precast SDS-PAGE gels (BioRad, Hercules, CA, USA) and run for approximately 1 h. Proteins were then transferred to a FL-PVDF membrane (Immobilon, Merck-Millipore, Burlington, MA, USA) and total protein stain was performed using Revert stain (LiCor Biotechnologies, Lincoln, NE, USA). Once the image for total protein was obtained, the membrane was decolored and blocked with a 2.5% BSA solution diluted in TBS buffer. The muHTT protein was detected using a primary antibody raised in rabbit, directed against the first 17 amino acids of HTT (N17) and a secondary anti-rabbit antibody, conjugated with IRDye800. Membranes were scanned on an Odyssey WB scanner.

### 2.10. Statistical Analysis

Statistical data analysis was undertaken using the GraphPad Prism (Version 8.4.3) software. One-way ANOVA followed by post hoc analyses (Sidak’s or Fisher’s LSD), or two-way ANOVA followed by Sidak’s or Dunnett’s test was performed as required. Alpha was set at 0.05 across all analyses.

## 3. Results

### 3.1. Leu-Fect Series of Transfection Reagents Exhibit Low Cellular Toxicity

**Figure 1:**
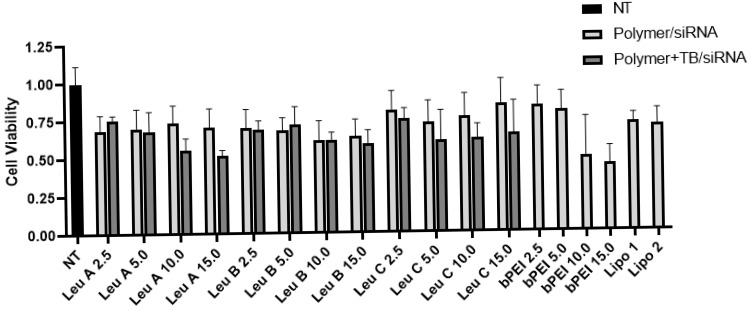
**Low toxicity of lipopolymers of the Leu-Fect series.** Naive N2a cells were treated with Leu-Fect A, B, and C complexed at various carrier/siRNA ratios with a non-targeting (scramble) siRNA, either in the presence or absence of Trans-Booster (ratio 1:1 to siRNA). After 48 h, cell viability was assessed using the MTT assay. Lipopolymers of the Leu-Fect series showed comparable levels of toxicity as Lipofectamine RNAiMax at ratios lipo/siRNA 1:1 and 2:1. Instead, bPEI shows increased toxicity at carrier/siRNA ratios of 10:1 and 15:1.

In previous studies, we showed how Leu-Fect series of transfection reagents can be used to safely deliver nucleic acids of distinct nature into in vitro cellular models of leukemia [26,27,28,29], as well as to xenograft tumors in vivo [30,31]. Moreover, we have studied the effects of Trans-Booster reagent, and we have previously shown that it contributed to the delivery of siRNAs to breast cancer cells [32]. However, the distribution of the siRNA complexes to the central nervous system (CNS), as measured by brain tissue levels of administered siRNA, was not significant. The question of whether Leu-Fect transfection reagents, including Trans-Booster reagent, can be potentially used to safely deliver nucleic acids into in vitro neuronal models, remains unanswered. One of the first issues we explored here is the relative sensitivity of neuronal cell lines to the toxicity of transfection reagents, including Leu-Fect lipopolymers. Thus, to test the potential toxicity of the Leu-Fect A, B, and C, we transfected naive N2a cells with 50 nM of control (scramble) siRNA, in complexes with Leu-Fect reagents at carrier/siRNA ratios ranging from 2.5:1 to 15:1, in the presence or absence of Trans-Booster (ratio 1:1 to siRNA). Relative cell viability was assessed 48 h later using the MTT assay. In all cases, there was a mild cellular toxicity, which seemed to increase with the carrier/siRNA ratio. Addition of the Trans-Booster additive to the complexes did cause a non-significant increase in toxicity across all lipopolymers. The commonly used branched bPEI carrier displayed more toxicity at the same carrier/siRNA ratios as compared to the Leu-Fect series of transfection reagents. The cytotoxicity of the Leu-Fect complexes with siRNA was on par with the well-established transfection reagent Lipofectamine^TM^ RNAiMax at the recommended ratios of 1:1 and 2:1, indicating that Leu-Fect series polymers could become an important tool for nucleic acids delivery into neuronal models without increased toxicity (Figure 1).

### 3.2. Leu-Fect Transfection Reagent Effectively Deliver siRNA into Neuronal Cells

**Figure 2:**
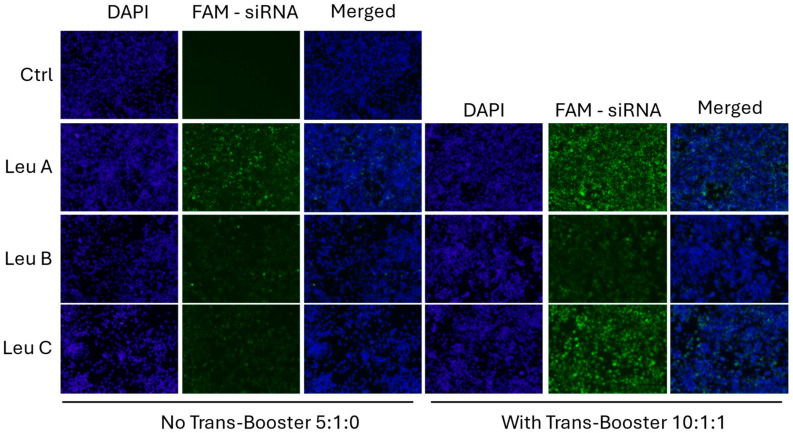
**Uptake of FAM-labeled siRNA by naive N2a cells when transfected using Leu-Fect polymers.** Naive N2a cells were treated with Leu-Fect A, B and C reagents complexed with a 40 nM of FAM-labeled siRNA in the presence or absence of Trans-Booster at different carrier:siRNA:Trans-Booster ratios. Internalization of FAM-siRNA is observed when using all three Leu-Fect reagents. Images obtained at 4×.

**Figure 3:**
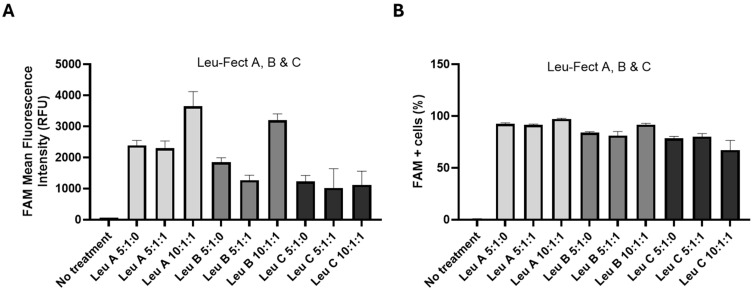
**Uptake of FAM-labeled siRNA by naive N2a cells when transfected using Leu-Fect polymers.** Naive N2a cells were treated with Leu-Fect A, B, and C reagents complexed with a FAM-labeled siRNA at different carrier/siRNA/Trans-Booster ratios. (**A**) MFI shows that all polymers were successfully delivering FAM-labeled siRNA into cells. Leu-Fect A and B showed a dose dependent effect. (**B**) Leu-Fect A, B, and C reagents were able to deliver FAM-labeled siRNA to 67–97% of naive N2a cells in culture.

Next, we assessed whether the Leu-Fect reagents could effectively deliver siRNA to the intracellular space of neuronal cells. For this, FAM-labeled control (scrambled) siRNA was complexed with Leu-Fect A, B, and C reagents, either at a 5:1 carrier/siRNA ratio when in absence of Trans-Booster, or 10:1:1 carrier/siRNA/Trans-Booster ratio. The naive N2a cells were then transfected with these polyplexes at 40 nM final siRNA concentration. After 24 h of transfection, cells exhibited positive intracellular fluorescence, indicating that FAM-siRNA was effectively delivered into the neuronal cells (Figure 2). To further quantify the efficacy of siRNA delivery, experiments were replicated with the carrier/siRNA/Trans-Booster ratios of 5:1:0, 5:1:1, and 10:1:1, and analyzed by flow cytometry. All Leu-Fect reagents at all ratios showed a significant population of FAM(+) fluorescent cells. However, Leu-Fect A and B showed a ratio-dependent increase in the mean fluorescence intensity (MFI), while Leu-Fect C did not. Moreover, addition of Trans-Booster to the polyplexes did not seem to cause a further increase in the observed MFI (Figure 3A). The percentage of FAM+ cells after transfection with Leu-Fect/FAM-siRNA polyplexes was robust, being higher in N2a cells transfected with Leu-Fect A, followed by Leu-Fect B and C, respectively. Once again, the addition of Trans-Booster did not seem to further increase the proportion of transfected cells (Figure 3B).

### 3.3. Leu-Fect/GFP siRNA Complexes Reduced muHTT Transcript and Chimeric Protein Levels

**Figure 4:**
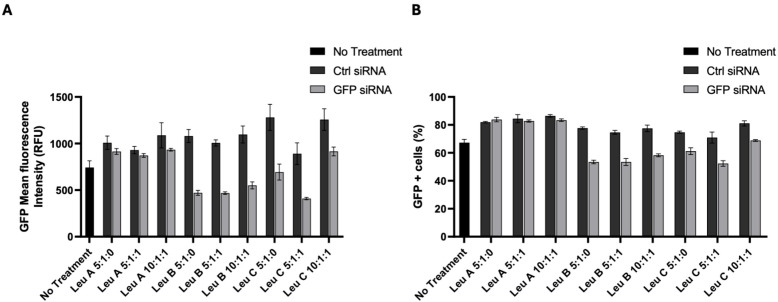
**Transfection of siRNA directed against GFP decreases muHTT-GFP fluorescence in N2a-97Q cells.** N2a-97Q cells were treated with Leu-Fect A, B, and C reagents complexed with a siRNA against the GFP portion of the transgene at different carrier/siRNA/Trans-Booster ratios. Analyses by flow cytometry show that Leu-Fect B and C, but not Leu-Fect A, can significantly decrease GFP MFI (**A**) and percentage of GFP-positive cells (**B**).

After confirming that Leu-Fect reagents effectively delivered the siRNA into the neuronal cells, we next tested whether this delivery also resulted in functional silencing of a target gene of interest. For this, we used N2a-97Q cells, stably expressing a construct encoding the mutated exon 1 (97 CAG repeats) of the human *HTT* gene, c-terminally tagged with eGFP. Then, a well-established siRNA targeting GFP mRNA [25,33,34] or a control (scramble) siRNA was complexed with the Leu-Fect reagents with or without Trans-Booster at carrier/siRNA/Trans-Booster ratios of 5:1:0, 5:1:1, and 10:1:1. The N2a-97Q cells were treated with these complexes for 48 h at 50 nM siRNA concentration, and then analyzed by flow cytometry. We hypothesized that, if the GFP section of the chimeric mRNA was effectively targeted and silenced with the GFP-specific siRNA, the expression of the whole chimeric construct will be accordingly decreased. The GFP siRNA complexes with Leu-Fect B and C effectively reduced the GFP fluorescence of N2a-97Q cells when compared to complexes made with Ctrl siRNA. This effect was not potentiated by the use of Trans-Booster (Figure 4A,B). In contrast, a minimal effect was observed with the Leu-Fect A/GFP siRNA complexes. We previously demonstrated that the affinity of Leu-Fect A for siRNAs was significantly stronger than the affinity of Leu-Fect B or C, suggesting that, although siRNA is effectively transported into the intracellular space of the cells by Leu-Fect A, it may not be promptly released from the complexes to be able to accomplish its silencing function [30].

To further confirm that the reduction in GFP fluorescence actually corresponded to a decrease in the whole chimeric muHTT-GFP mRNA, we proceeded to check the abundance of the transcript by RT-qPCR, using primers that would align with the 5′ extreme (corresponding to the N-terminus of the protein) of the chimeric mRNA. One forward primer and three different reverse primers were designed. Two of the reverse primers bind within the muHTT exon 1 sequence, while the third reverse primer binds to the GFP section of the mRNA (Supplementary Figure S1A,B). The N2a-97Q cells were transfected with Leu-Fect B complexed with GFP siRNA (50 nM) for 48 h. Upon total RNA purification and cDNA synthesis, the qPCR analysis indicated a decrease in the expression of the whole chimeric transcript when silenced with an siRNA against the GFP section of the fusion gene. Moreover, two out of three sets of primers designed to bind the N17 section of the mRNA show consistent silencing (Supplementary Figure S1C).

**Figure 5:**
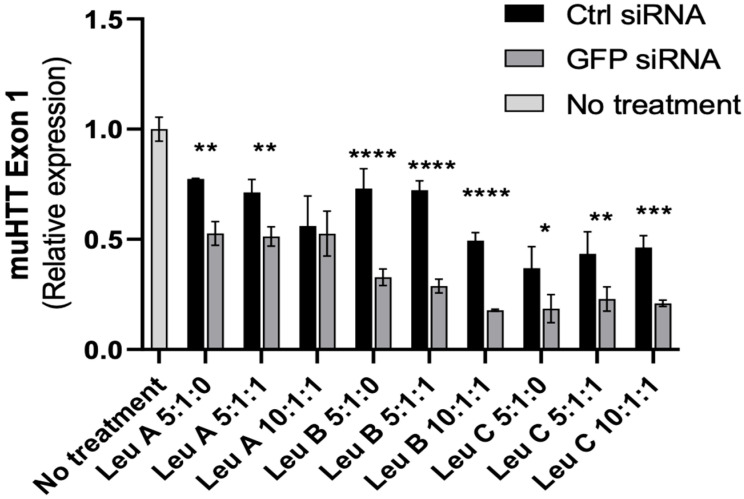
**siRNA directed against GFP delivered with Leu-Fect polymers silences transcription of muHTT-GFP in N2a-97Q cells.** N2a-97Q cells were treated with Leu-Fect A, B, and C reagents complexed with a siRNA against the GFP portion of the transgene. RT-PCR using primers that span the muHTT region show effective silencing of the transgene. * *p* < 0.05; ** *p* < 0.01; *** *p* < 0.001; **** *p* < 0.0001. Two-way ANOVA and Sidak’s multiple comparison test.

**Figure 6:**
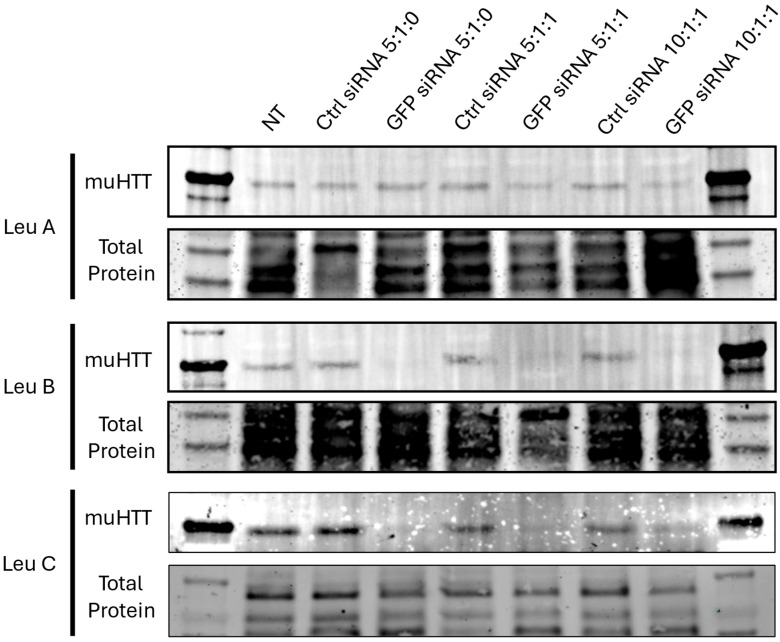
**GFP siRNA delivered using Leu-Fect B and C polymers decrease the amount of soluble muHTT-GFP chimeric protein.** N2a-97Q cells were treated with Leu-Fect reagents complexed with GFP siRNA at different carrier/siRNA/Trans-Booster ratios. The WB demonstrated the effect of Leu-Fect B and C/GFP siRNA complexes (but not Leu-Fect A complexes) substantially reducing the amount of soluble muHTT. Original scanning images are shown in Supplementary Figure S2.

Once a set of viable qPCR primers was identified, we proceeded to test the relative efficacy of the Leu-Fect reagents for silencing chimeric muHTT-GFP mRNA. As observed previously by the flow cytometry analysis, Leu-Fect B and C were far superior to Leu-Fect A in silencing the transcription of the chimeric gene (Figure 5). Furthermore, this silencing caused by Leu-Fect B and C matched a decrease in the amount of chimeric protein detected by Western blot, using an antibody that specifically recognizes the N17 N-terminus domain of HTT (Figure 6). Consistent with previous findings, Leu-Fect A did not show a strong effect on the amount of chimeric protein (indicating less efficiency than the other reagents). Once again, addition of Trans-Booster did not seem to cause a positive effect in the silencing efficiency.

**Figure 7:**
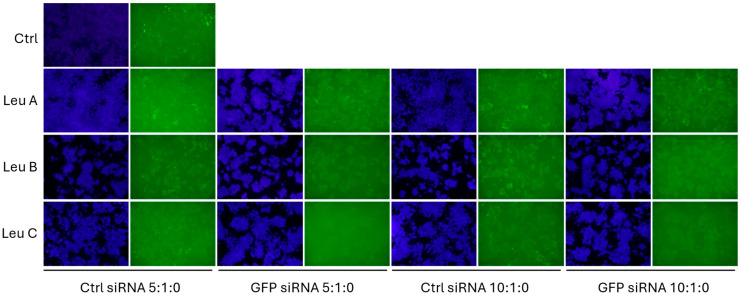
**Transfection of siRNA directed against GFP decreases the amount of aggregated mutant huntingtin in N2a-97Q cells.** N2a-97Q cells were treated with Leu-Fect A, B and C polymers complexed with a siRNA against the GFP portion of the transgene. Images obtained at 4×.

**Figure 8:**
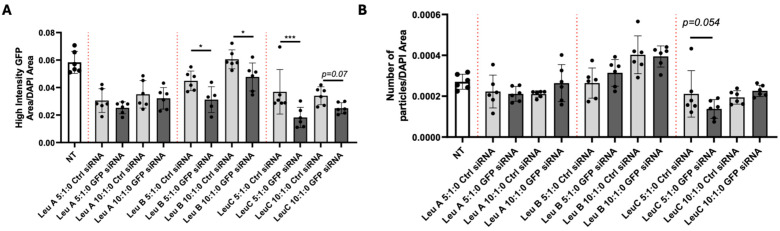
**Image analysis of amount of aggregated mutant huntingtin in N2a-97Q cells after transgene silencing.** N2a-97Q cells were treated with Leu-Fect A, B, and C reagents complexed with a siRNA against the GFP portion of the transgene. (**A**) The total punctate GFP area normalized over the DAPI-positive area was decreased when the transgene was silenced using Leu-Fect B and C complexes. (**B**) Number of particles/DAPI area. * *p* < 0.05; *** *p* < 0.001. One-Way ANOVA with Fisher’s LSD multiple comparisons.

One of the hallmarks of HD is the aggregation of muHTT in neuronal cells, and it is believed that this continuous process of oligomerization and aggregation is at least partially responsible for the striatal and cortical neuronal death so characteristic of HD [5,35,36,37]. Animal models of the disease, such as the R6/2 mice [38,39,40] replicate the phenotype as well. In culture, a subset of N2a-97Q cells contain a punctuated pattern of high-intensity GFP fluorescence that resemble the muHTT aggregates observed in humans and animal models of the disease. We wondered whether the silencing effect of Leu-Fect reagents complexed with the GFP-siRNA would have an effect on the size or the number of said puncta. For this, the N2a-97Q cells were transfected with Leu-Fect reagents complexed with either Ctrl or GFP siRNAs (50 nM) at 5:1 and 10:1 carrier/siRNA ratios. No Trans-Booster was utilized since it did not show any potential advantage in this specific model. After 48 h, cells were fixed, and nuclei were stained with DAPI for fluorescence microscopy (Figure 7). Image analyses show that the area of high-intensity GFP puncta, normalized by total DAPI area, was significantly reduced in cells treated with complexes made with GFP siRNA and Leu-Fect B and C, but not with Leu-Fect A, in comparison with complexes made with Ctrl siRNA (Figure 8A). However, the total number of particles over the DAPI only slightly decreased with Leu-Fect C at 5:1 ratio (Figure 8B), suggesting that silencing of muHTT-GFP might not only prevent the growth or the expansion of already existing aggregates, but may have limited effect on aggregates that are already formed.

### 3.4. Leu-Fect C/HTT siRNAs Complexes Reduce muHTT Burden in Neuronal Cells

Having shown that the Leu-Fect reagents could deliver the siRNAs intracellularly in neuronal cells and that a siRNA targeting the GFP motif of a chimeric protein also shows silencing of the transgene, we next utilized three different siRNAs directed against the 5′ portion of the chimeric gene, corresponding to the exon 1 (N-terminus) of HTT protein. Two of these sequences were based on previously published studies [23,24], and one of them was generated using common bioinformatic tools for siRNA design (Supplementary Figure S3).

**Figure 9:**
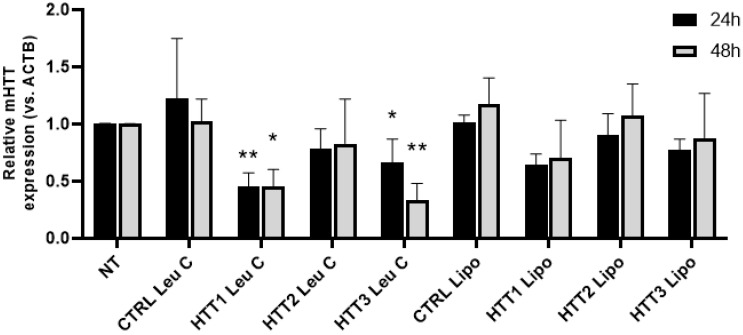
**Screening of HTT siRNA sequences.** N2a-97Q cells were treated with Leu-Fect C polymer complexed with three distinct siRNAs (carrier/siRNA ratio 5:1) directed against different positions on the HTT portion of the transgene. Cells were also treated with siRNAs complexed with Lipofectamine^TM^ RNAiMax at a ratio of 2:1. The siRNAs 1 and 3 have higher efficacy, and this is more evident when using Leu-Fect C polymers as compared to the Lipofectamine^TM^ RNAiMax. * *p* < 0.05; ** *p* < 0.01. Two-way ANOVA and Dunnett’s multiple comparison test.

**Figure 10:**
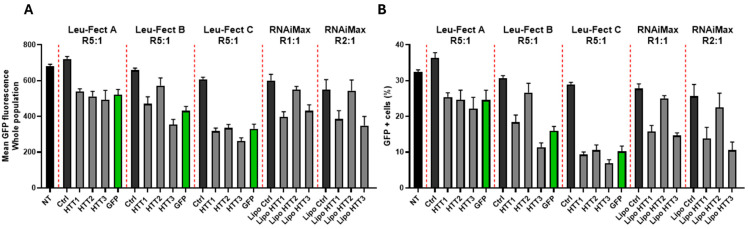
Transfection of siRNA directed against HTT section of transgene decreases muHTT-GFP fluorescence in N2a-97Q cells. N2a-97Q cells were treated with Leu-Fect A, B, and C reagents complexed with three siRNA candidates against the HTT portion of the transgene at a 5:1 carrier/siRNAratio. Leu-Fect B and C showed a significant decrease in MFI (**A**) and percentage of GFP+ cells (**B**). Representative histograms are shown in Supplementary Figure S4.

First, we used Leu-Fect C complexes with either Ctrl siRNA or HTT siRNAs to assess silencing after 24 and 48 h. We also tested the siRNAs by using Lipofectamine^TM^ RNAiMAX. The siRNAs HTT1 and HTT3, but not HTT2, showed significant silencing at both timepoints. Interestingly, although we observed a trend towards silencing using these two siRNAs with Lipofectamine^TM^ RNAiMAX, this did not reach statistical significance (Figure 9). Then, we assessed whether the silencing effect of Leu-Fect C observed at the mRNA level could be translated to the protein level, as well as to other members of the Leu-Fect series. We transfected N2a-97Q cells with the HTT siRNAs as well as with GFP siRNA, using Leu-Fect reagents at a ratio 5:1. For comparison, we also delivered the HTT siRNAs using Lipofectamine^TM^ RNAiMAX at ratios 1:1 and 2:1. Forty-eight hours after transfection, cells were analyzed by flow cytometry. As previously observed, HTT1 and HTT3 siRNAs were the most efficacious siRNAs. The combination of HTT1 and HTT3 siRNAs with Leu-Fect C showed the highest reduction in fluorescence, followed by Leu-Fect B and Leu-Fect A, respectively. The efficacy of HTT1 and HTT3 siRNA was on par with the GFP siRNA previously used (Figure 10A,B). Interestingly, the efficacy of HTT1 and HTT3 siRNAs was also observed when using Lipofectamine^TM^ RNAiMAX as the delivery vehicle (Figure 10A,B).

**Figure 11:**
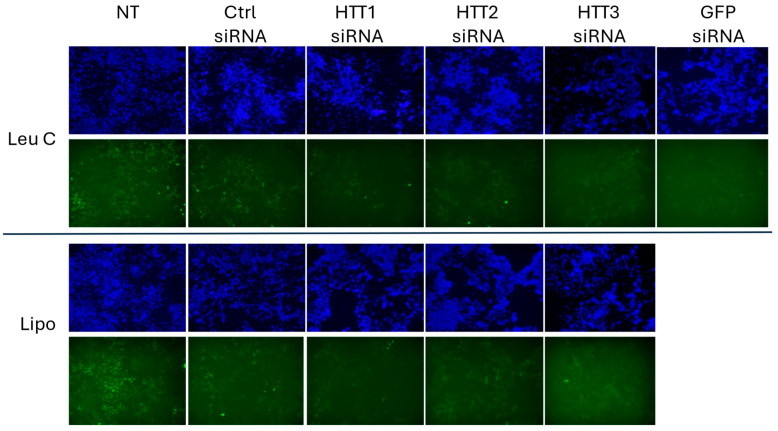
Transfection of siRNA directed against HTT section of transgene using Leu-Fect C decreases the amount of aggregated mutant huntingtin in N2a-97Q cells. N2a-97Q cells were treated with Leu-Fect C polymer complexed with control, GPF, and three siRNA candidates against the muHTT portion of the transgene at a 5:1 carrier/siRNA ratio (**Top**). Control and HTT1-3 siRNAs were delivered using LipofectamineTM RNAiMax at a 2:1 carrier/siRNA ratio (**Bottom**). Representative pictures are shown. Images obtained at 4×.

**Figure 12:**
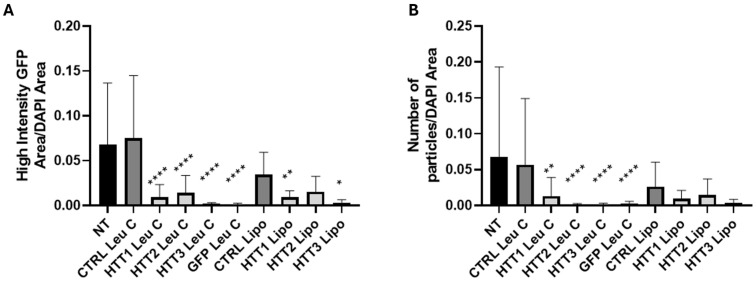
Transfection of siRNA directed HTT section of transgene decreases the amount of aggregated mutant huntingtin in N2a-97Q cells. N2a-97Q cells were treated with Leu-Fect C polymer complexed with a siRNA against the HTT portion of the transgene at a 5:1 carrier/siRNA ratio. (**A**) The total punctate GFP area normalized over the DAPI-positive area. (**B**) Number of GFP particles normalized over the DAPI-positive area. * *p* < 0.05; ** *p* < 0.01; **** *p* < 0.0001. One-way ANOVA and Sidak’s multiple comparisons test.

Lastly, we assessed whether *HTT* silencing using Leu-Fect C to deliver HTT siRNAs would also have an effect on the muHTT aggregates, as observed with previous GFP siRNA (Figure 11). Leu-Fect C complexes with all HTT and GFP siRNAs were able to significantly reduce the area of high-intensity GFP puncta normalized by total DAPI area, as well as the number of GFP high-intensity particles. The siRNAs delivered with Lipofectamine^TM^ RNAiMAX also exhibited a reduction in the high-intensity GFP-positive area and number of particles, but this outcome was more modest in comparison to the Leu-Fect C complexes (Figure 12A,B).

## 4. Discussion

This study demonstrates successful optimization of a lipopolymer-based siRNA delivery system (Leu-Fect reagents) for reducing muHTT expression in an in vitro HD model. Initially, the siRNA uptake was confirmed using a fluorescently labeled siRNA, demonstrating the lipopolymers of the Leu-Fect series have the capability to deliver siRNA effectively, as shown by fluorescent microscopy. When using flow cytometry in an attempt to quantify the uptake, we noticed striking differences between the three lipopolymers. For instance, Leu-Fect A and B seem to show classical dose–response behavior, while Leu-Fect C does not. This could be due to a multiple of reasons. Our methods allow us to only observe one snapshot in the continuous balance between uptake and metabolism. For instance, it was previously shown that Leu-Fect A possesses a higher affinity than Leu-Fect B and C for siRNA [30], and these differences may contribute, not only to the uptake but also to the length of time that a siRNA is present in the cells, considering its metabolism. Further experiments will be required to address these potential confounding factors.

Initial screening with GFP siRNA confirmed that Leu-Fect B and C exhibited enhanced cellular uptake, effective transgene silencing, and low cytotoxicity. A slight transcriptional effect was observed when cells were treated with Ctrl siRNA paired with Leu-Fect lipopolymers, when compared to untreated samples, which might indicate an early sign of toxicity (or non-specific effect) that is not observed by the MTT assay. Of notice, the Trans-Booster additive did not seem to increase efficacy, but it shows a non-significant trend in increased toxicity, especially at high doses. It is possible that the anionic nature of the Trans-Booster additive may have synergistic effects at the level of the plasma membrane, mildly increasing toxicity, without contributing to intracellular uptake of siRNA.

When Leu-Fect C lipopolymer was paired to a Huntingtin-specific siRNA, as it was the best candidate with the initial silencing studies with GFP siRNA, it achieved the highest efficacy and significantly reduced the HTT mRNA levels. Most notably, this formulation using an HTT-siRNA not only decreased the size of perceived pathogenic aggregates, but also prevented their expansion, most likely as a result of a halt in further muHTT aggregation due to a decreased availability of soluble muHTT protein. This highlights the potential of the Leu-Fect C/HTT-siRNA combination for a therapeutic intervention in HD. Since the current study was focused on in vitro feasibility studies, an important aspect of the siRNA delivery, namely inflammatory features, were not fully explored. However, in a previous study, we have shown that the lipopolymers in an in vitro model of cultured human peripheral blood mononuclear cells (PBMCs), induce minimal secretion of cytokines such as TNF-α, IL-6, and IFN-γ [41]. This indicates that the lipopolymers of the Leu-Fect series may not likely trigger a disproportionate inflammatory responses in preclinical animal models, but this hypothesis is yet to be tested.

In the context of HD therapeutic strategies, our findings align with recent advancements in siRNA-based interventions. Khvorova’s group developed a unimolecular ‘dual-targeting’ divalent siRNA scaffold capable of co-silencing two genes in the CNS, achieving significant and sustained silencing of both target genes for at least two months post-administration in mice [42]. Mathews et al. demonstrated that transcriptional repression of *muHTT* via nuclear-acting HTT-lowering methods like antisense oligonucleotides, CRISPR-Cas9 genome editing, Lac repressor systems, and zinc finger proteins effectively reduced somatic instability, whereas cytoplasmic-acting treatments like siRNA-mediated HTT reduction did not influence somatic instability, indicating that direct transcriptional suppression is crucial for modulating repeat expansions [43]. These studies underscore the potential of advanced siRNA scaffolds and DNA-targeted approaches in modulating multiple gene targets within the CNS. Furthermore, although siRNA-bearing lipid nanoparticles (LNPs) have been investigated for neurological disorders, to our knowledge there are only a handful of studies for their direct use for HTT mRNA targeting in HD models. In one study, the researchers utilized LNP-mediated delivery of unlocked nucleic acid modified siRNA targeting CAG repeats to selectively suppress polyQ-expanded proteins, including muHTT in mouse models. This approach effectively reduced the muHTT levels in CNS without affecting the wild-type allele at a higher dose of 1.8 ug of LNP-siRNA [44]. Our research contributes to this growing body of evidence by demonstrating the efficacy of Leu-Fect lipopolymers in effectively delivering siRNA to reduce mutant HTT expression, highlighting the promise of non-viral delivery systems in HD treatment. An additional noteworthy finding in this study was the enhanced efficacy of Leu-Fect lipopolymers over the commercial Lipofectamine^TM^ reagent in reducing HTT mRNA levels and mutant Huntingtin’s aggregates in N2a-97Q cells. This improved performance aligns with previous research demonstrating the effectiveness of Leu-Fect lipopolymers in delivering various siRNAs and mRNAs in both in vitro and in vivo settings [27,45].

These findings have important implications for advancing siRNA-based therapeutics for HD. Effective gene silencing of muHTT is a crucial therapeutic strategy; however, challenges such as limited cellular uptake and potential off-target toxicity have hindered clinical translation [46]. For instance, antisense oligonucleotides administered at concentrations of 200 nM or higher have been associated with nonspecific impacts on protein synthesis in vitro. Similarly, siRNAs can induce off-target effects that are dose-dependent. Notably, siRNAs have been observed to cause unexpected and varying changes in the levels of non-targeted proteins in mammalian cells [47]. Moreover, certain siRNAs can trigger toxic phenotypes in a manner independent of their intended targets, underscoring the importance of careful siRNA design and dosing to minimize such adverse outcomes [48]. Although off-target effects are innate to siRNA therapies, we believe that an improved biocompatibility, the higher transfection efficiency of Leu-Fect C at relatively low doses, paired with a directed injection/delivery to the target organ, in this case the CNS, could overcome these barriers, providing a more efficient and safer delivery platform for siRNA therapy. Additionally, the observed reduction in both the size and number of muHTT aggregates aligns with previous studies showing that early intervention can mitigate disease progression and neuronal toxicity [49]. These results support further investigation into lipopolymer-based delivery systems as a promising alternative to viral vectors or chemically modified siRNAs currently in development.

From the experiments conducted in this study, it can be said that these studies emphasize the efficacious transfection reagents and how different polymer structures have such drastic effects at penetrating the cell membrane and achieving gene silencing effects. Despite the promising outcomes of our lipopolymer-based siRNA delivery system, several challenges remain to be addressed. To start, we used a chimeric construct comprising the exon 1 of human muHTT tagged with GFP, which was used as a surrogate assessment that may not represent the effect our vehicles/siRNAs on full-length muHTT. For that, new cellular models or patient-derived cell lines will be studied in the upcoming experiments. Additionally, while our in vitro results are encouraging, the in vivo stability of the described carriers and their ability to effectively cross the BBB require further investigations. The BBB presents a daunting obstacle in delivering therapeutics to the CNS, and ensuring that our delivery system can traverse this barrier without compromising its integrity is crucial for clinical application. Various strategies such as opening of tight junctions, receptor-mediated transcytosis, cell-mediated transport, carrier-mediated transport, and adsorptive-mediated transcytosis have been explored to facilitate nanoparticle passage across the BBB [50]. Having demonstrated the utility of Leu-Fect lipopolymers in vitro, future studies should focus on testing these siRNA formulations in cells derived from HD patients, such as primary fibroblasts or induced pluripotent stem cell (iPSC)-derived neurons. This approach would enhance external validity by confirming that the polyplexes are effective in human cells rather than only in mouse neuronal models. Finally, identifying an optimal, efficient, and noninvasive route of administration for siRNA delivery is crucial, as previous studies using Leu-Fect reagents in leukemia models have demonstrated limited siRNA biodistribution to the brain [30]. Incorporating focused ultrasound (FUS) with microbubble contrast agents has emerged as a promising noninvasive method for delivering siRNA across the BBB in HD models. Burgess et al. demonstrated that FUS-induced BBB disruption facilitates targeted siRNA delivery to the striatum, resulting in significant, dose-dependent reductions in Huntington (Htt) mRNA levels [51]. Therefore, alternative delivery routes, such as intrastriatal or intrathecal administration, should be explored. Addressing these challenges is essential for translating our findings into viable therapeutic strategies for Huntington’s disease.

## Figures and Tables

**Table 1:** Sequence of the sense and antisense strands of siRNAs used.

siRNA	Sense Strand	Antisense Strand	Region of HTT (Nucleotides)
HTT1 siRNA	UUCAUCAGCUUUUCCAGGGdTdC	CCCUGGAAAAGCUGAUGACdGdG	8–26
HTT2 siRNA	GCCUUCGAGUCCCUCAAUdCdC	ACUUGAGGGACUGAAGGCdCdT	28–44
HTT3 siRNA	UUGCUGUUGCUGCUGUUGGdAdA	CCAACAGCAGCAACAGCAAdCdA	49–71 (in CAG mutation)
GFP-22 siRNA	GAACUUCAGGGUCAGCUUGCCG	GCAAGCUGACCCUGAAGUUCAU	620–644
Ctrl siRNA	AUGCUACCAAUUCAAGUGAAGAUdTdA	UAAUCUUCACUUGAAUUGGUAGCAUUU	N/A

## Data Availability

Data is contained within the article and Supplementary Materials.

## Supplementary Materials

The following supporting information can be downloaded at: https://www.mdpi.com/article/10.3390/pharmaceutics17060726/s1.
