# Supplementary material for: In Vitro Efficacy of PEI-Derived Lipopolymers in Silencing of Toxic Proteins in a Neuronal Model of Huntington’s Disease
Source: Pharmaceutics. 2025 May 30;17(6):726. doi: 10.3390/pharmaceutics17060726 (PMC12196245; doi:10.3390/pharmaceutics17060726)

# In Vitro Efficacy of PEI-Derived Lipopolymers in Silencing of Toxic Proteins in a Neuronal Model of Huntington's Disease

Luis C. Morales[1,4#], Luv Modi[1], Saba Abbasi Dezfouli[1], Amarnath Rajendran[1,2], Remant KC[1,2], Vaibhavi Kadam[3,4], Simonetta Sipione[3,4], Hasan Uludağ[1,2]

## Supplementary Figures

**Supplementary Figure S1: Primer design and testing for the detection of muHTT transcript. A)** One forward primer and three reverse primers were designed for the quantification of the muHTT-GFP transcript. Schematic representation of binding sites of the primers. **B)** Sequences of the tested primers **C)** N2a-97Q cells were treated with Leu-Fect B polymer complexed with a siRNA against the GFP portion of the transgene at different carrier:siRNA:Trans-Boosterratios. Primers FWD with Rev1 and Rev2 are able to amplify muHTT cDNA, as well as detect the silencing caused by lipopolymer siRNA complexes.

**A**

| Name | Sequence |
|---|---|
| N17-Fwd | GACCCTGGAAAAGCTGATGA |
| N17-Rev1 | TTGGAAGCTTTTGAGGGACT |
| N17-Rev2 | TGGAAGCTTTTGAGGGACTC |
| GFP-Rev3 | AACAGCTCCTCGCCCTTG |

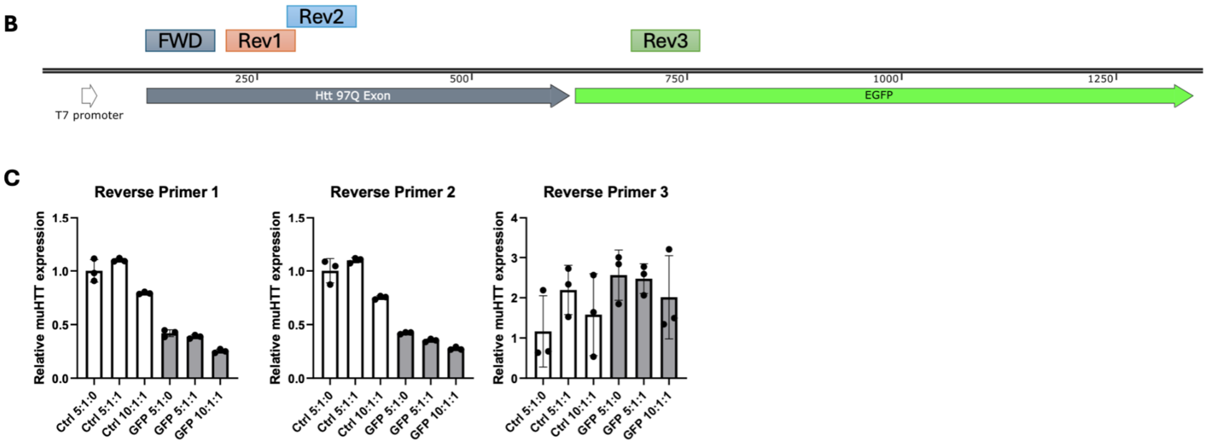

Supplementary Figure S2: Full scanning of Western blot images shown in Figure 6 of the main manuscript. Top: Leu-Fect A; Middle: Leu-Fect B; Bottom: Leu-Fect C. For all gels lanes were loaded in the following order: MWM; No treatment; Control siRNA 5:1:0; GFP siRNA 5:1:0; Control siRNA 5:1:1; GFP siRNA 5:1:1; Control siRNA 10:1:1; GFP siRNA 10:1:1; MWM

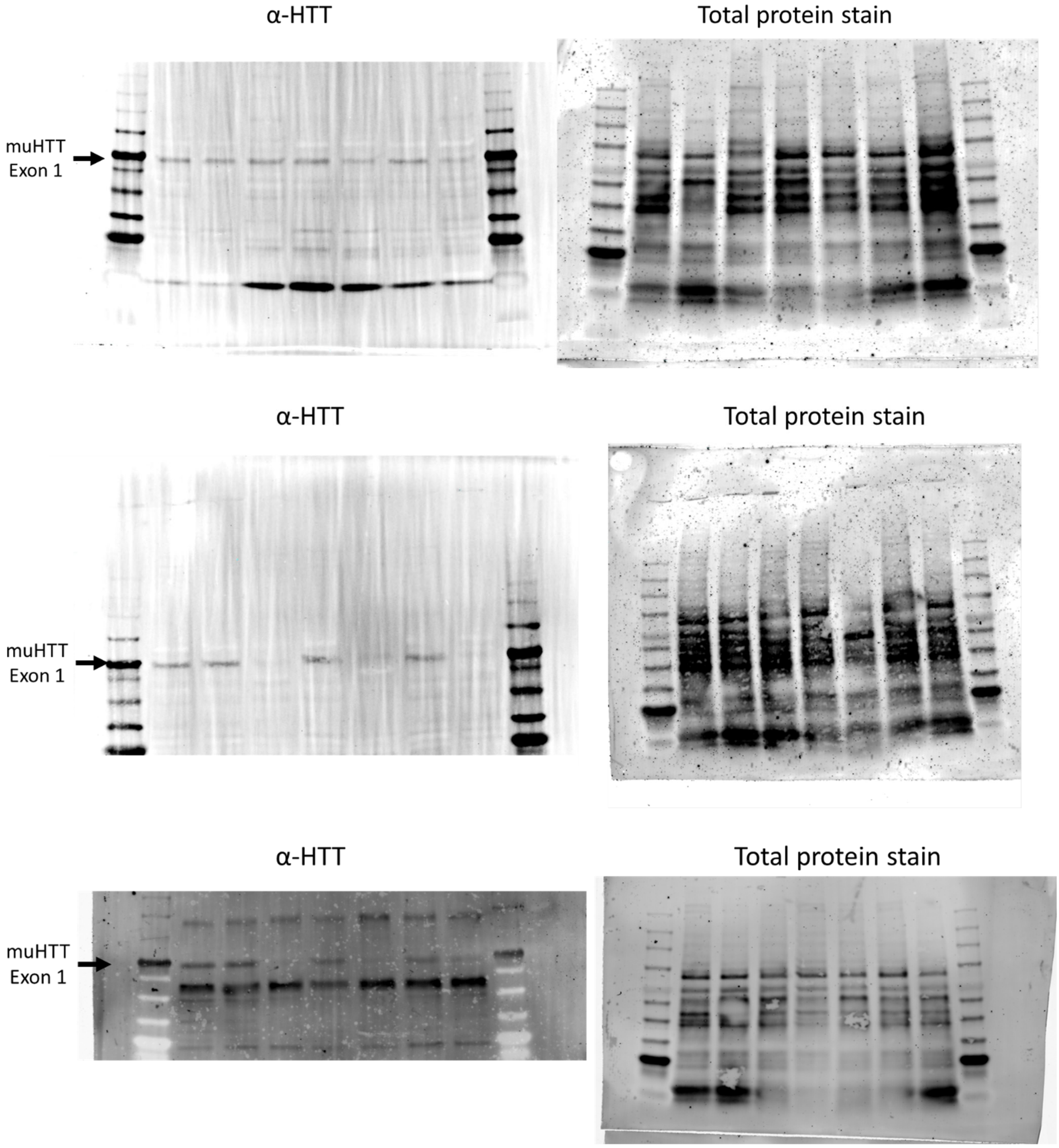

Supplementary Figure S3: Diagram of binding sites for the muHTT and GFP siRNAs.

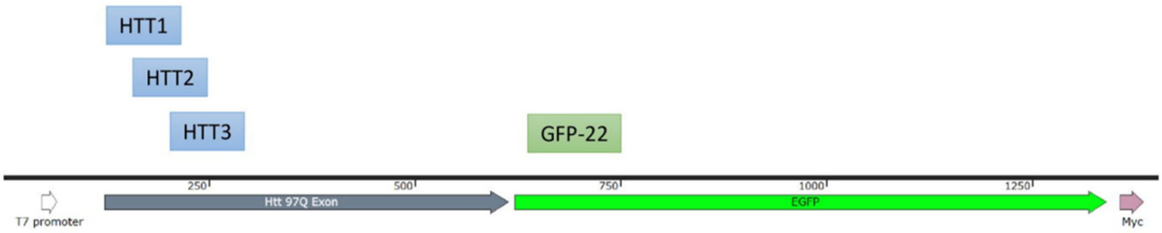

Supplementary Figure S4. Representative flow cytometry histograms from cells treated with Leu-Fect lipopolymers complexed with either Ctrl siRNA or siRNA targeted against the N17 portion of the muHTT-GFP transgene

Non-fluorescent N2a cells:

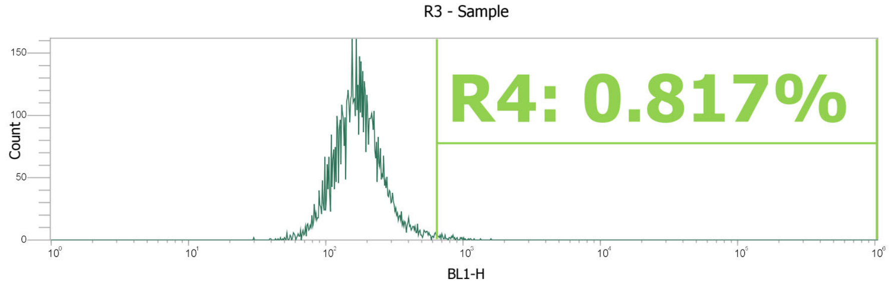


Non-treated N2a-97Q cells:

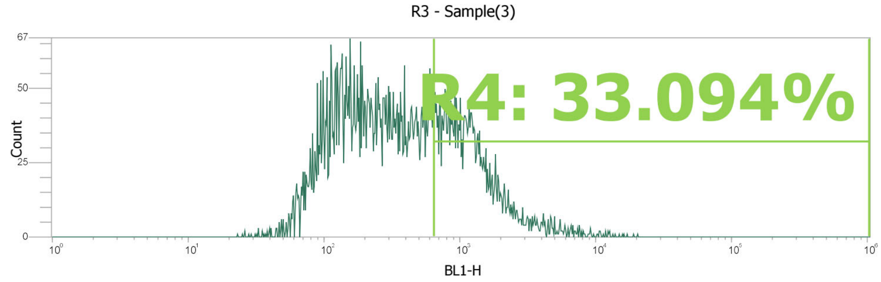


Leu-Fect A – Ctrl siRNA

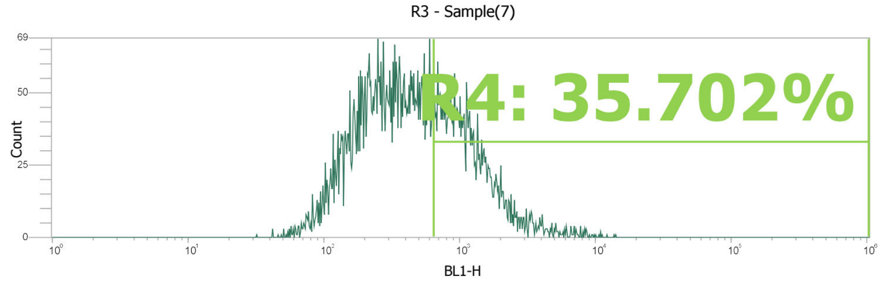


Leu-Fect A – HTT1 siRNA

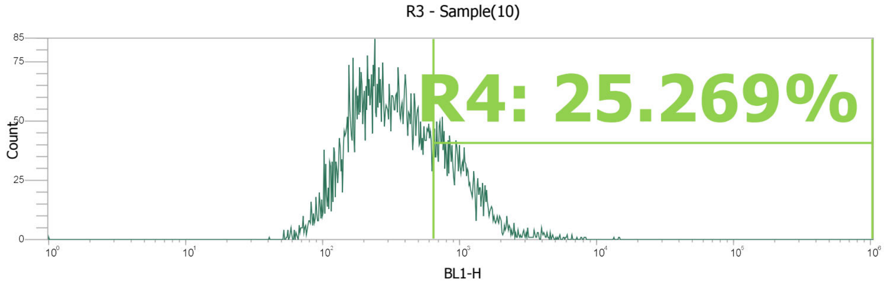

Leu-Fect A – HTT2 siRNA

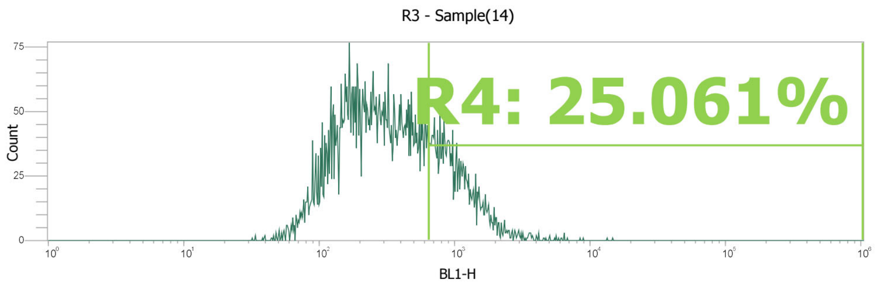


Leu-Fect A – HTT3 siRNA

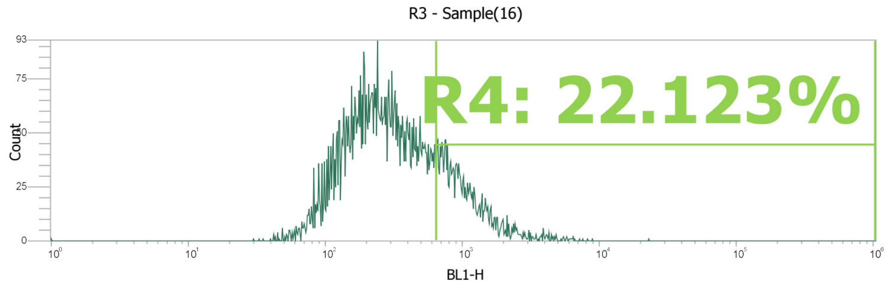


Leu-Fect A – GFP siRNA

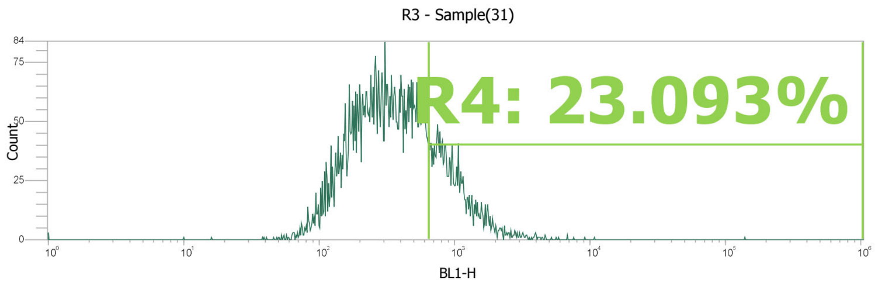


Leu-Fect B – Ctrl siRNA

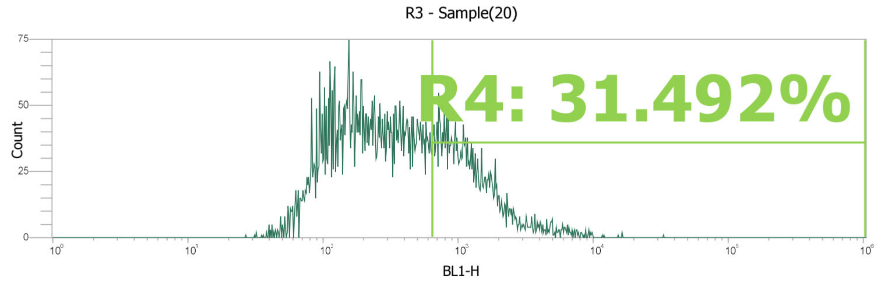

Leu-Fect B – HTT1 siRNA

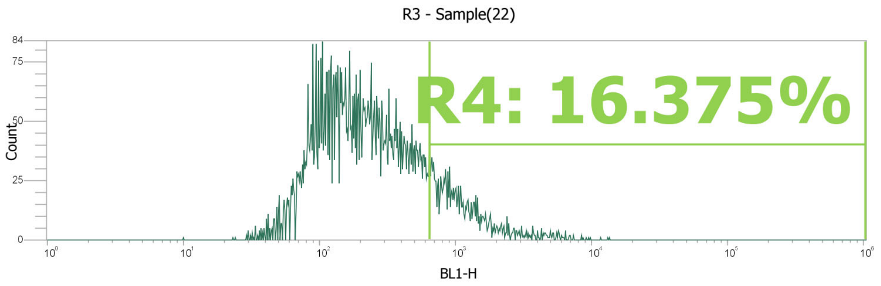


Leu-Fect B – HTT2 siRNA

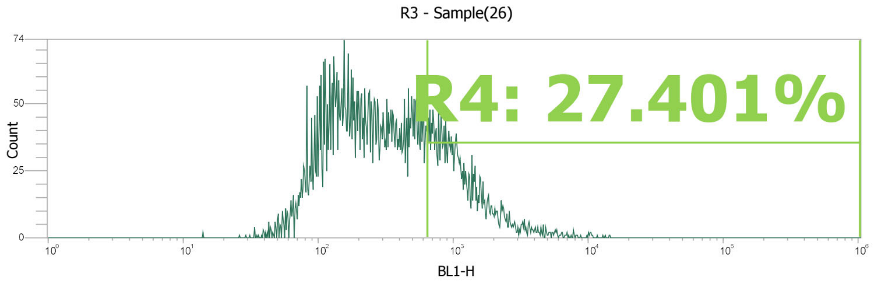


Leu-Fect B – HTT3 siRNA

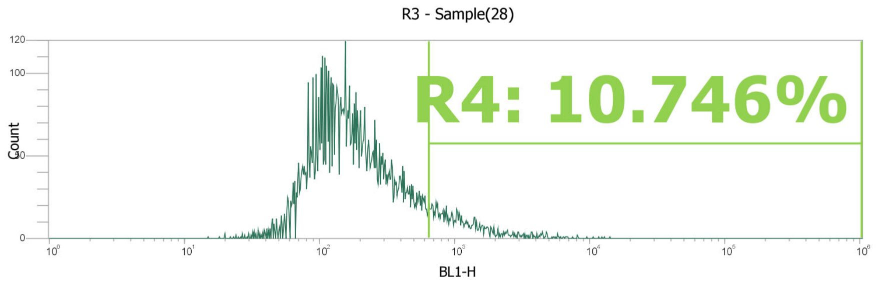


Leu-Fect B - GFP siRNA

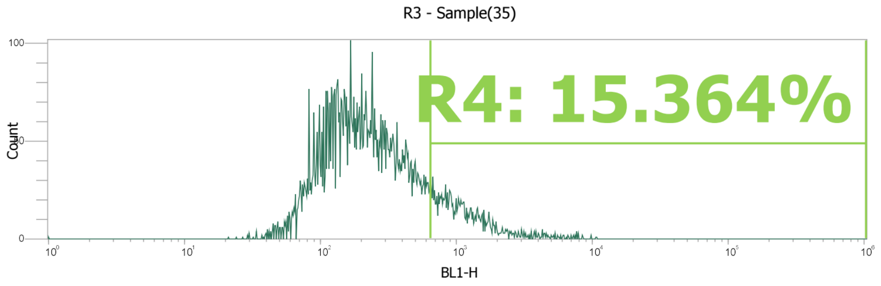

Leu-Fect C – Ctrl siRNA

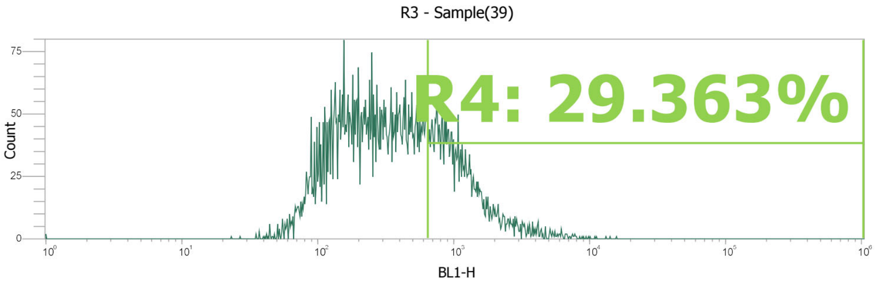


Leu-Fect C – HTT1 siRNA

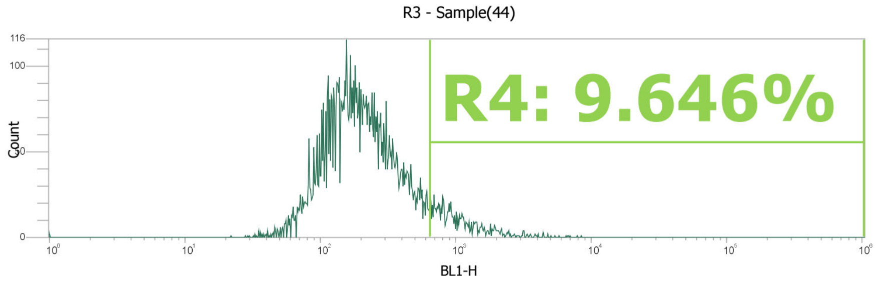


Leu-Fect C – HTT2 siRNA

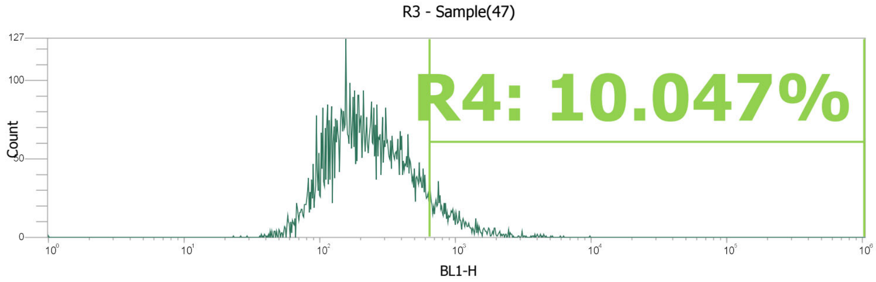


Leu-Fect C – HTT3 siRNA

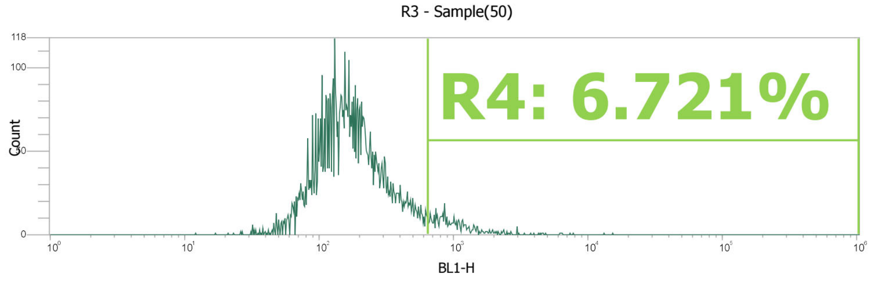

Leu-Fect C – GFP siRNA

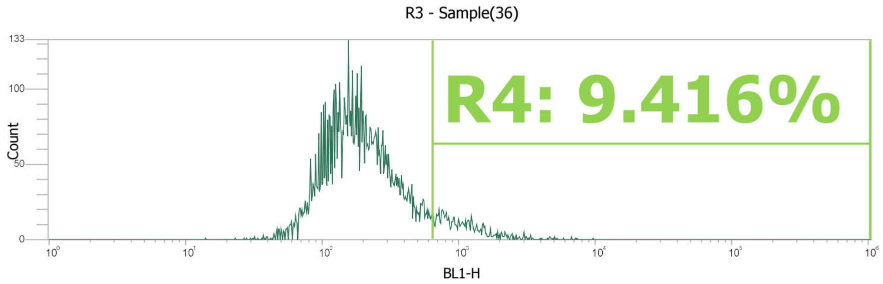


Lipofectamine – Ctrl siRNA

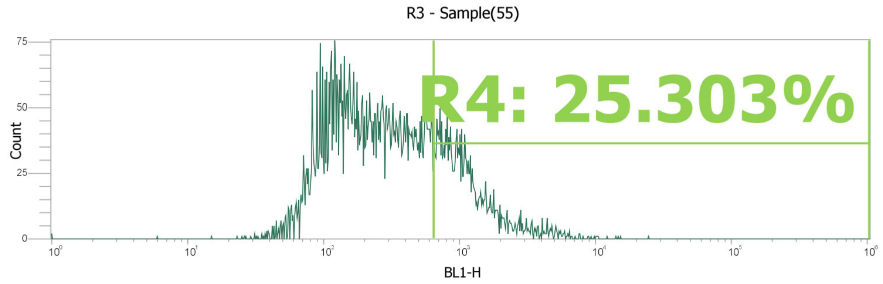


Lipofectamine – HTT1 Ratio 1:1

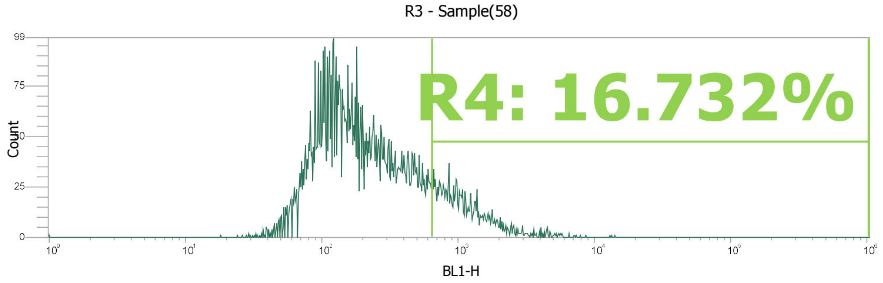


Lipofectamine – HTT2 Ratio 1:1

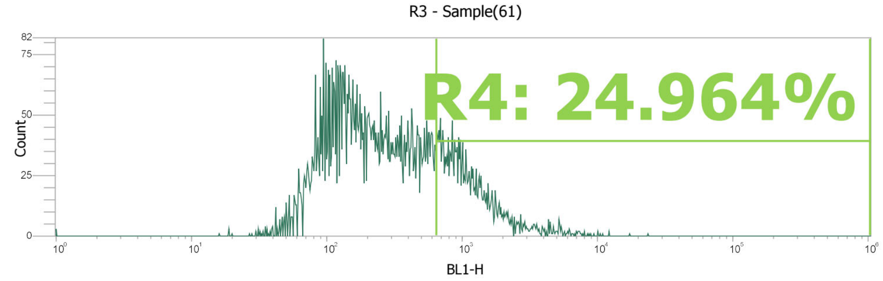

Lipofectamine – HTT3 Ratio 1:1

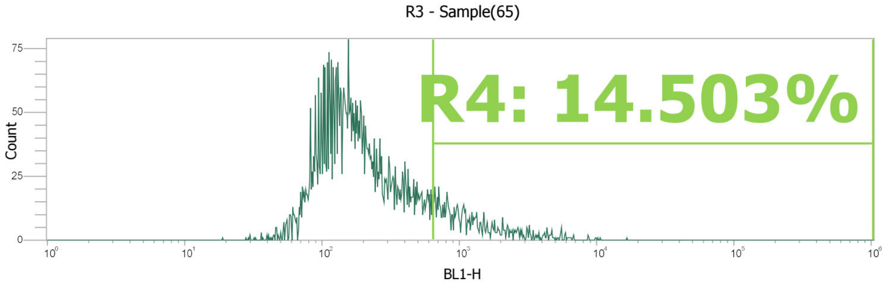


Lipofectamine – HTT1 Ratio 2:1

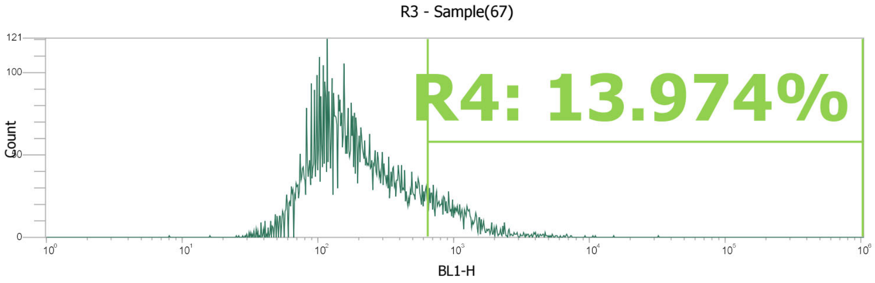


Lipofectamine – HTT2 Ratio 2:1

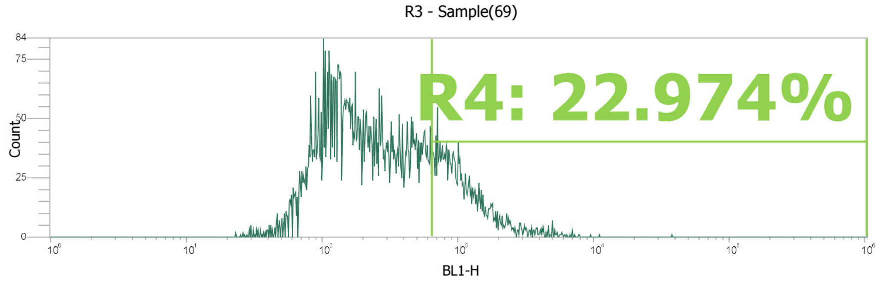


Lipofectamine – HTT3 Ratio 2:1

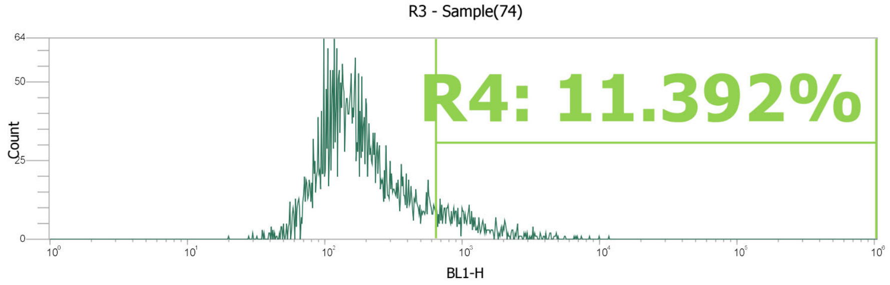

Supplement: Supplementary file 1 [file pharmaceutics-17-00726-s001.zip › pharmaceutics-3597527-supplementary.pdf]
